# A Coupled Calibration Method for Dual Cameras-Projector System with Sub-Pixel Accuracy Feature Extraction

**DOI:** 10.3390/s24061987

**Published:** 2024-03-20

**Authors:** Ran Jia, Junpeng Xue, Wenbo Lu, Zeyu Song, Zhichao Xu, Shuxin Lu

**Affiliations:** School of Aeronautics and Astronautics, Sichuan University, Chengdu 610065, China

**Keywords:** 3D shape measurement, projector calibration, binocular, phase target

## Abstract

Binocular structured light systems are widely used in 3D measurements. In the condition of complex and local highly reflective scenes, to obtain more 3D information, binocular systems are usually divided into two pairs of devices, each having a Single Camera and a Projector (SCP). In this case, the binocular system can be seen as Dual Cameras-Projector (DCP) system. In the DCP calibration, the Left-SCP and Right-SCP need to be calibrated separately, which leads to inconsistent parameters for the same projector, thus reducing the measurement accuracy. To solve this problem and improve manoeuvrability, a coupled calibration method using an orthogonal phase target is proposed. The 3D coordinates on a phase target are uniquely determined by the binocular camera in DCP, rather than being calculated separately in each SCP. This ensures the consistency of the projector parameters. The coordinates of the projector image plane are calculated through the unwrapped phase, while the parameters are calibrated by the plane calibration method. In order to extract sub-pixel accuracy feature points, a method based on polynomial fitting using an orthogonal phase target is exploited. The experimental results show that the reprojection error of our method is less than 0.033 pixels, which improves the calibration accuracy.

## 1. Introduction

Three-dimensional shape measurement technology is of great significance in many applications, such as intelligent manufacturing, industrial inspection, virtual reality, machine vision, reverse engineering, biomedicine and so on [1,2,3]. Among all of three-dimensional shape measurement methods, Fringe Projection Profilometry (FPP) has been widely studied due to its advantages of being non-contact and high-speed and having high accuracy, high spatial resolution and a large field of view [4,5,6]. In FPP systems, the Digital Light Processing (DLP) projector is commonly used for its advantages of being low cost and having flexible programming [7]. Two typical configurations of the FPP system are the Single Camera-Projector (SCP) system [8,9,10,11] and the Dual Cameras-Projector (DCP) system [12,13].

In the SCP system, the projector is equal to a camera. Therefore, this kind of FPP system can also be regarded as a binocular vision system in principle, which then can be described by the binocular vision model. In the DCP system, the projector projects groups of phase-shifting fringe patterns onto the objects, and the modulated fringe patterns are captured by the binocular camera. Typically, projectors are used to provide a binocular camera with easily matched features [14]. In this case, it is not necessary to calibrate the parameters of the projector.

However, when meet the condition of covering the complex surface between different views or local high reflection, the binocular camera is often unable to capture some unfavorable feature points at the same time, which will result in a matching failure. In this way, in order to further improve the measurement accuracy in difficult scenes, many researchers have regarded the projector as a camera [14,15,16,17], to ensure that as long as one camera in the binocular camera can capture the feature point, the measurement can be realized. Tao et al. [18] use the constraints of the multi-view system, projecting fringes embedded with triangular waves onto objects, in order to retrieve absolute phase and high-speed dynamic 3D measurements of isolated objects. Liu et al. [15] proposed a stereo matching method without phase unwrapping. This method uses the three view geometric constraints of the camera and the projector, which can effectively reduce the number of fringes in the binocular structured light system. Gai et al. [16] use the digital projector to provide additional information for multi-view mapping, which can effectively avoid the problems of small field of view and self-occlusion in 3D measurement. Hu et al. [17] proposed an accurate dynamic 3D shape measurement method based on DIC-assisted phase shifting and a stereo structured-light system model, which requires projected three-step phase-shifting patterns and a speckle pattern. All of those methods require accurate calibration of the projector.

Existing projector calibration methods can be divided into two categories. In the first way, the camera captures both the calibration chessboard and the projected chessboard in the same scene [19,20]. These methods use the calibrated parameters of the camera to calculate the parameters of the projector so that the calibration error of the camera will be accumulated and amplified in the process of projector calibration.

To avoid projector calibration being affected by the calibration errors of the camera, some researchers proposed the “inverse camera” method. The fringe patterns are generated and projected onto the calibration target, and the pixel coordinates of the feature points on the image plane of the projector are determined. In the “inverse camera” method, the camera is just used to enable the projector to “capture” the calibration target. The precision of these methods depends on the accuracy of extracting and mapping the corresponding pixel coordinates of feature points on the image plane of the camera and projector. In [9], the phase-shifting patterns are directly projected onto the printed chessboard, and feature points are extracted by the camera and mapped to their pixel coordinates on the image plane of the projector. Zhang et al. [21] implemented a sub-pixel mapping between the corresponding feature points on the image plane of the camera and the projector, which was based on the projection invariance of the cross ratio. At the same time, with the popularization of machine learning and deep learning, some learning-based calibration methods have also been emerged. Liu et al. [22] proposed a Bayesian network according to the Markov random field hypothesis, which transforms the image intersection point matching problem between a camera and the projector into a maximum a posteriori estimation problem.Yuan et al. [23] designed an unsupervised image deblurring network to recover a sharp target image from the deteriorated one, which can learn more accurate features from the multi-quality target dataset of convenient image acquisition.

Nevertheless, these methods are only applicable to projector calibration under SCP systems. Among the existing multi-view reconstruction methods [15,16,17,18], the Left-SCP and Right-SCP are generally calibrated separately in the system calibration stage. This will cause inconsistencies in the projector parameters, which are mainly reflected in the focal length error and principal point error. These errors can lead to reconstruction errors and rigid transformations and rotations, resulting in a decrease in measurement accuracy. Through the calculation and derivation of the formula, it can be quantitatively calculated that the principal point error will not only cause the reconstruction error, but also cause the rigid body transformation of the 3D data. At the same time, focal length errors can also cause reconstruction errors, and can also cause the 3D data to rotate around one axis.

Another key point in the projector calibration is the extraction accuracy of the feature points. Classical feature points include chessboard corners and circular target centers [8,21,24,25,26]. Xing et al. [24] proposed a method for calibrating the measurement system which has lens distortions. Fitting the phase values projected on the chessboard through a rational function, the phase value of the corner is accurately extracted, and then the corresponding pixel coordinates of the corner on the image plane of the projector are determined. Since the corners of the chessboard are sensitive to light, this method has low accuracy and reliability. Huang et al. [25] proposed a sub-pixel extraction method based on the circle pattern. A group of pixels at the edge of the circle are extracted and mapped to the image plane of the projector. Then the center of the circle is fitted through the least-square method. Due to the perspective projection of the camera, the center of the fitted circle is usually not the center of the true circle target [27]. Chen et al. [26] proposed an improved camera and projector calibration method, an improved sub-pixel edge detection algorithm and a circular projection error compensation algorithm.

Most of the existing methods require high-precision targets with good diffuse reflection. Such targets can provide reliable world coordinates to ensure the accuracy of calibration results. In addition, these methods only apply to SCP systems, corresponding to the problem of inconsistent projector parameters mentioned above.

**Figure 1:**
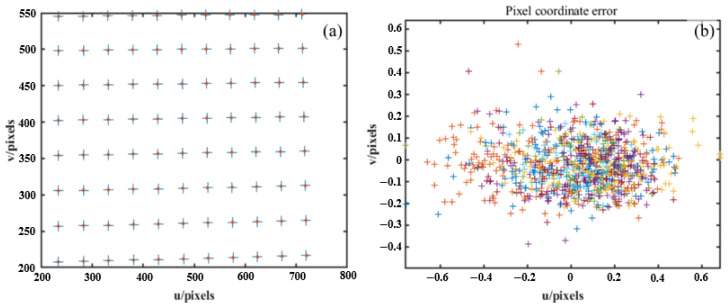
Pixel coordinate errors. (**a**) Projector pixel coordinates of a certain pose. (**b**) Reprojection error distribution of different poses.

Ideally, if we calibrate each SCP system in the DCP system independently, the calibrated projector parameters should be consistent. However, due to the extraction error and phase error, there are differences between those projector pixel coordinates corresponding to the same feature point, which is calculated through different pairs of projector-cameras. Figure 1a shows the projector pixel coordinates of a certain pose calculated by two pairs of SCPs. The STD of two direction errors are 0.1548 pixels and 0.1045 pixels, respectively. Figure 1b shows the reprojection errors of each target pose, while each pose is represented by a unique color cross symbol. It can be observed that due to the error mentioned above, the calibrated projector parameters of the two pairs of SCPs are different. This will inevitably cause errors in the 3D measurement results.

This paper proposes a coupled projector calibration method for the DCP system. Through the binocular camera in DCP system and the orthogonal fringe map, our method can obtain the relationship between the 3D coordinates in the world coordinate system and the 2D coordinates on the image plane of the projector, solving the inconsistencies of the projector parameters. Moreover, our method can obtain high-precision projector parameters without the high-precision chessboard targets or circular targets.

The rest of the paper is organized as follows. Section 2 explains the related work about the proposed calibration method. Section 3 introduces the pipeline and details of our methods. Section 4 gives the experimental results to demonstrate the effectiveness of the method. Section 5 discusses the innovation of this study. Section 6 summarizes this paper.

## 2. Related Works

### 2.1. Camera Model and Projector Model

The camera model is the simplification of optical imaging geometry, and the pinhole model is widely used because of its simplicity and accuracy [28]. Let the 3D point in the world coordinate system be $W(x_{w},y_{w},z_{w})$, while its homogeneous coordinates are $\tilde{W}\left(x_{w},y_{w},z_{w},1\right)$. In the following, the subscript *c* represents the camera model. Let the corresponding point in the camera image coordinate system be $w_{c}\left(u_{c},v_{c}\right)$, while its homogeneous coordinates are ${\tilde{w}}_{c}\left(u_{c},v_{c},1\right)$. The relationship between the world coordinates and the camera image coordinates can be described as

(1) $$s_{c}\left[\begin{array}{c} u_{c}\\ v_{c}\\ 1 \end{array}\right]=\left[\begin{array}{ccc} f_{cu} & \gamma_{c} & u_{c0}\\ 0 & f_{cv} & v_{c0}\\ 0 & 0 & 1 \end{array}\right]\left[\begin{array}{cc} R_{c} & T_{c} \end{array}\right]\left[\begin{array}{c} x_{w}\\ y_{w}\\ z_{w}\\ 1 \end{array}\right]=A_{c}\left[\begin{array}{cc} R_{c} & T_{c} \end{array}\right]\left[\begin{array}{c} x_{w}\\ y_{w}\\ z_{w}\\ 1 \end{array}\right]$$

where $f_{cu}=f_{c}/d_{cu}$ and $f_{cv}=f_{c}/d_{cv}$. $f_{c}$ is the focal length of the camera lens, while $d_{cu}$ and $d_{cv}$ are the pixel size along the *u* and *v* axes, respectively. $\left(u_{c0},v_{c0}\right)$ is the coordinate of the principal point. $\gamma_{c}$ is the skew factor, $s_{c}$ is an arbitrary scale factor and $A_{c}$ is the intrinsic matrix. $R_{c}$ and $T_{c}$ denote the 3 × 3 rotation matrix and the 3 × 1 translation vector from the world coordinate system to the camera image coordinate system, respectively. The matrix composed of $R_{c}$ and $T_{c}$ is the extrinsic matrix.

Due to the distortion of the camera lens, the actual camera model is often not an ideal pinhole model. Through the distortion model, we can obtain the correct correspondence between 3D space points and 2D pixel points. The most commonly used distortion model is the Brown–Conrady model [29], which mainly contains two kinds of distortions: radial distortion and tangential distortion. Let the ideal image point be $\left(x_{c},y_{c}\right)$, and the corresponding distorted point be $\left(x_{d}^{c},y_{d}^{c}\right)$; the relationship between them can be described as

(2) $$\left\{\begin{array}{c} x_{d}^{c}=x_{c}\left(1+k_{c1}{r_{c}}^{2}+k_{c2}{r_{c}}^{4}\right)+\left[2p_{c1}x_{c}y_{c}+p_{c2}\left({r_{c}}^{2}+2{x_{c}}^{2}\right)\right]\;\\ y_{d}^{c}=y_{c}\left(1+k_{c1}{r_{c}}^{2}+k_{c2}{r_{c}}^{4}\right)+\left[p_{c1}\left({r_{c}}^{2}+2{y_{c}}^{2}\right)+2p_{c2}x_{c}y_{c}\right] \end{array}\right.$$

where $\left(k_{c1},k_{c2}\right)$ denotes the radial distortion coefficients, $\left(p_{c1},p_{c2}\right)$ denotes the tangential distortion coefficients and ${r_{c}}^{2}={x_{c}}^{2}+{y_{c}}^{2}$ is satisfied. The high-order coefficient term is discarded because its distortion value is insignificant.

The intrinsic matrix $A_{c}$ and the distortion coefficients $\left(k_{c1},k_{c2},p_{c1},p_{c2}\right)$ are constant parameters, while the extrinsic matrix $\left[\begin{array}{cc} R_{c} & T_{c} \end{array}\right]$ varies with the poses of the calibration target. Through single-camera calibration, the intrinsic parameters and the distortion coefficients of the camera can be determined.

The projector can be regarded as an inverse camera, so it can also be modeled by the pinhole model with radial and tangential lens distortion. The formula for describing the projector model is the same as Equations (1) and (2), except that the subscript needs to be replaced from *c* to *p*.

### 2.2. Phase Target

Phase targets are widely used in camera calibration because of their robustness against defocussing and their flexible feature points [30,31,32,33]. Differing from the traditional inverse camera method based on the diffuse planar target, the method based on the phase target only depends on the horizontal and vertical fringe patterns to obtain the feature points. These methods avoid extracting the complex feature points (such as the corner points of the chessboard, the center of the circle or the cross line, etc.). In addition, theoretically, all points distributed on the phase target can be used as effective 2D calibration points, so the amount of 2D calibration points is greatly increased, while the calibration accuracy is also improved. Moreover, while under the certain calibration accuracy, the number of required 2D calibration planes can be reduced, and the calibration process can be simplified. The following will introduce the phase-shifting method to obtain the horizontal and vertical phases. A set of horizontal and vertical fringe patterns is generated by computer and projected by projector. The vertical fringe patterns captured by the camera can be expressed as

(3) $$I_{Vn}\left(u_{c},v_{c}\right)=A_{V}\left(u_{c},v_{c}\right)+B_{V}\left(u_{c},v_{c}\right)\cos\left[\varphi_{V}\left(u_{c},v_{c}\right)+\frac{2\pi n}{N}\right],\;n=1,2,\ldots ,N-1$$

The horizontal fringe patterns captured by the camera can be expressed as

(4) $$I_{Hn}\left(u_{c},v_{c}\right)=A_{H}\left(u_{c},v_{c}\right)+B_{H}\left(u_{c},v_{c}\right)\cos\left[\varphi_{H}\left(u_{c},v_{c}\right)+\frac{2\pi n}{N}\right],\;n=1,2,\ldots ,N-1$$

where $\left(u_{c},v_{c}\right)$ is the pixel coordinate on the camera image plane, $A_{V}\left(u_{c},v_{c}\right)$ and $A_{H}\left(u_{c},v_{c}\right)$ are the vertical and horizontal background intensities. $B_{V}\left(u_{c},v_{c}\right)$ and $B_{H}\left(u_{c},v_{c}\right)$ are the vertical and horizontal modulation intensities. $\varphi_{V}\left(u_{c},v_{c}\right)$ and $\varphi_{H}\left(u_{c},v_{c}\right)$ are the vertical and horizontal phase values modulated by the height of the object. The subscript *n* is the sequence number of the group of fringe pattern images, while *N* represents the total number of steps of the fringe phase-shifting. The phase value can be calculated by the following formula

(5) $$\varphi_{j}\left(u_{c},v_{c}\right)=-\arctan\frac{\sum_{n=0}^{N-1}I_{jn}\left(u_{c},v_{c}\right)\sin\left(\frac{2\pi n}{N}\right)}{\sum_{n=0}^{N-1}I_{jn}\left(u_{c},v_{c}\right)\cos\left(\frac{2\pi n}{N}\right)},\;\;j=V,H$$

The value of $\varphi_{j}\left(u_{c},v_{c}\right)$ is wrapped in the range of (−π,π] by Equation (5). To obtain the continuous phase value $\varphi_{V}\left(u_{c},v_{c}\right)$ and $\varphi_{H}\left(u_{c},v_{c}\right)$, the phase unwrapping algorithm is needed to eliminate the 2π phase discontinuity. In this paper, the multi-frequency time phase unwrapping algorithm [34] is selected to obtain the corresponding unwrapped phase.

Much research shows that as *N* increases, the phase-shifting method will have better anti-noise performance, while the precision of the obtained fringes and the quality of the phases will also improve [35]. Therefore, in this paper, we chose an eight-step method, instead of the commonly used four-step phase-shifting method.

## 3. Calibration Method

### 3.1. Overview

**Figure 2:**
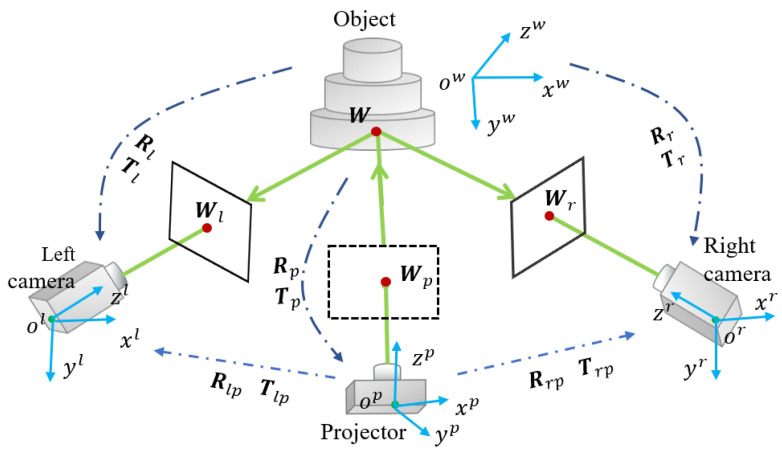
DCP system.

The DCP system is always set up as shown in Figure 2. $o^{w}-x^{w}y^{w}z^{w}$, $o^{w}-x^{l}y^{l}z^{l}$, $o^{r}-x^{r}y^{r}z^{r}$ and $o^{p}-x^{p}y^{p}z^{p}$ denote the world, left camera, right camera and projector coordinate systems, respectively. The relationship between camera, projector and world coordinates can be described as

(6) $$\left\{\begin{array}{c} W=R_{l}W_{l}+T_{l}\\ W=R_{r}W_{r}+T_{r}\\ W=R_{p}W_{p}+T_{p} \end{array}\right.$$

where W, $W_{l}$, $W_{r}$ and $W_{p}$ are the same points defined in $o^{w}-x^{w}y^{w}z^{w}$, $o^{w}-x^{l}y^{l}z^{l}$, $o^{r}-x^{r}y^{r}z^{r}$ and $o^{p}-x^{p}y^{p}z^{p}$, respectively. $R_{l}$, $R_{r}$ and $R_{p}$ denote the rotation matrices from the world to two cameras and projector; $T_{l}$, $T_{r}$ and $T_{p}$ denote the translation vectors. Uniting any two formulas in Equation (6) can solve the three-dimensional coordinates of the target point in the world coordinate system. In Equation (6), $R_{l}$, $T_{l}$, $W_{l}$, $R_{r}$, $T_{r}$ and $W_{r}$ can be determined by Zhang’s method [28], and $R_{p}$, $T_{p}$ and $W_{p}$ can be determined by the projector calibration proposed by our method. In addition, it is necessary to find the pose relationship between the left and right cameras and the projector for the system calibration.

Set the projector coordinate system as the reference, then eliminate the world coordinate W in Equation (6) to obtain

(7) $$\left\{\begin{array}{c} W_{p}=R_{p}^{-1}R_{l}W_{l}+R_{p}^{-1}\left(T_{l}-T_{p}\right)=R_{lp}W_{l}+T_{lp}\;\\ W_{p}=R_{p}^{-1}R_{r}W_{r}+R_{p}^{-1}\left(T_{r}-T_{p}\right)=R_{rp}W_{r}+T_{rp} \end{array}\right.$$

where $R_{p}^{-1}R_{l}$ and $R_{p}^{-1}\left(T_{l}-T_{p}\right)$ are the rotation matrix and translation vector between the left camera coordinate system and the projector coordinate system, denoted as $R_{lp}$ and $T_{lp}$. Likewise, $R_{p}^{-1}R_{r}$ and $R_{p}^{-1}\left(T_{r}-T_{p}\right)$ are the rotation matrix and translation vector between the right camera coordinate system and the projector coordinate system, denoted as $R_{rp}$ and $T_{rp}$.

**Figure 3:**
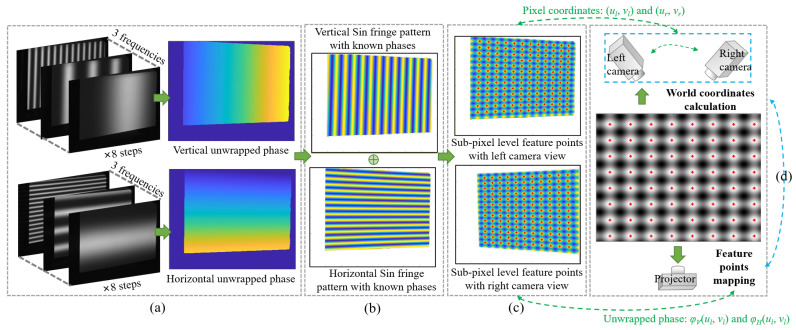
The pipeline of projector calibration. (**a**) Eight-step phase shift and Three-frequency heterodyne for phases unwrapping. (**b**) Create the orthogonal fringe map for feature extraction. (**c**) Extract the feature points on both right and left image. (**d**) Optimize the reprojection error.

In order to solve $R_{p}$, $T_{p}$ and $W_{p}$, we proposed a method to calibrate the projector in the DCP system. The complete calibration procedure with the proposed method can be summarized in the following steps:

- Step 1: Calibrate the intrinsic and extrinsic parameters of the two cameras;
- Step 2: Project two sets of fringe patterns, one horizontal and the other vertical, onto a white plane. Capture the images of these fringe patterns, respectively, with the two cameras calibrated in step 1;
- Step 3: Randomly change the poses of the white plane, then repeat step 1 and step 2 to obtain 17 groups of images. Each group contains 96 images; the left and right cameras correspond to 48 pictures each. For each camera, 8 vertical fringe patterns and 8 horizontal fringe patterns with three frequencies are need;
- Step 4: As shown in Figure 3a, for each group of images, calculate the absolute phase maps from the vertical and horizontal fringe patterns obtained by the binocular camera in DCP system;
- Step 5: Create the orthogonal fringe map for feature extraction as Figure 3b and extract the feature points on both right and left images as Figure 3c with the method given in Section 3.2. Compute the projector pixel coordinates and the world coordinates of each feature point with the method given in Section 3.2 and Section 3.3;
- Step 6: Estimate the intrinsic parameters and the distortion coefficients of the projector by optimizing the reprojection error with the Levenberg–Marquardt method as shown in Figure 3d.

The following part will introduce the details about our method.

### 3.2. Feature Points Extraction and Mapping

**Figure 4:**
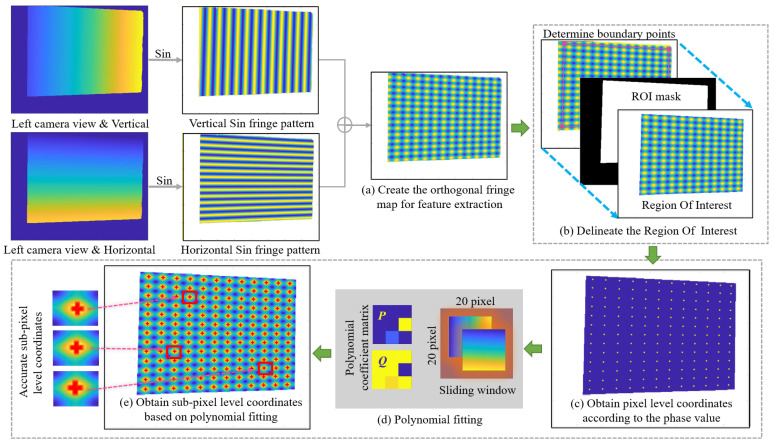
The process of detecting feature points.

Theoretically, any phase value can be used as a feature point, which is also the advantage of using phase targets, i.e., with a large number of accurate feature points. However, the complete period phase is more convenient for calculating the coordinates of feature points on the projector image plane. Other work that utilizes phase targets, such as [31], also uses the complete period phase as feature points. In order to facilitate the extraction and analysis, we select the phase value with a complete period as the feature points. After obtaining the unwrapped phases, the vertical and horizontal sine fringe patterns can be generated using the known phases, and then superimposed onto the orthogonal fringe map, as shown in Figure 4a. In this way, it is easier to capture the feature points. We select the intersections of the orthogonal bright fringe patterns to be feature points. Points with phase values of $\varphi_{V}^{tar}=2\pi n_{i}$ and $\varphi_{H}^{tar}=2\pi n_{j}$ are selected as feature points, where $n_{i}$ and $n_{j}$ are integral numbers. Since the accuracy of projector calibration often depends on the accuracy of feature point extraction, consequently, in order to obtain higher accuracy, we proposed a method based on polynomial fitting to extract the feature points with sub-pixel level coordinates.

Since there is some background information, the image needs to be pre-processed as follows. As shown in Figure 4b, take the left view as an example. Select four boundary points and calculate the ROI (Region of Interest) mask based on them. Boundary points are determined by hand-selecting the intersections of bright fringes and refining them using the same method as the polynomial fitting described below. This ensures that all feature points in the ROI have complete phase information. Finally, delineate the ROI. In the following process, we are only interested in the complete feature points in the ROI.

In the process of detecting feature points, we first obtain the pixel level coordinates of the orthogonal intersections according to the phase value of pixels like Figure 4c. Then we use the fitting method to further obtain sub-pixel coordinates. Because the white board in our method cannot be treated as an ideal plane, it may be tilted or have subtle unevenness properties, which can cause the phase growth to change from linear to nonlinear. The polynomial fitting method can better fit the geometric characteristics of an ordinary white board, so as to obtain more accurate phase information. Therefore, we choose the polynomial fitting method but not the usual plane fitting method as shown in Figure 4d. We set a sliding window with the size of 20 × 20 pixels as a sub-region for each integer pixel. Based on the least squares method, we use the integer pixel values and their phases in the sub-region to fit the polynomial equation, which can describe the distribution of horizontal and vertical unwrapped phases. The equation is as follows

(8) $$\left\{\begin{array}{c} p_{0}x^{2}y^{2}+p_{1}xy^{2}+p_{2}y^{2}+p_{3}x^{2}y+p_{4}xy+p_{5}y+p_{6}x^{2}+p_{7}x+p_{8}=u_{c}^{p}\;\\ q_{0}x^{2}y^{2}+q_{1}xy^{2}+q_{2}y^{2}+q_{3}x^{2}y+q_{4}xy+q_{5}y+q_{6}x^{2}+q_{7}x+q_{8}=v_{c}^{p} \end{array}\right.,\;\left\{\begin{array}{c} x=\varphi_{V}^{c}\\ y=\varphi_{H}^{c} \end{array}\right.$$

where $u_{c}^{p}$ and $v_{c}^{p}$ are the pixel level integer pixel coordinates in the sliding window, $\varphi_{V}$ and $\varphi_{H}$ are the corresponding unwrapped phases and $p_{n}\left(n=0,1,\ldots ,8\right)$ and $q_{n}\left(n=0,1,\ldots ,8\right)$ are the polynomial coefficients. Then convert the coefficients into the form of matrix for easier calculation, as P and Q show in the following formula

(9) $$P=\left[\begin{array}{ccc} p_{0} & p_{3} & p_{6}\\ p_{1} & p_{4} & p_{7}\\ p_{2} & p_{5} & p_{8} \end{array}\right],\;\;Q=\left[\begin{array}{ccc} q_{0} & q_{3} & q_{6}\\ q_{1} & q_{4} & q_{7}\\ q_{2} & q_{5} & q_{8} \end{array}\right]$$

Sub-pixel coordinates can be obtained by bringing the target phase $\varphi_{V}^{tar}=2\pi n_{i}$ and $\varphi_{H}^{tar}=2\pi n_{j}$ into Equation (8). The process of obtaining sub-pixel level coordinates can be expressed as

(10) $$\left\{\begin{array}{c} u_{c}^{sp}=f\left(P,\varphi_{V}^{tar},\varphi_{H}^{tar}\right)\;\\ v_{c}^{sp}=f\left(Q,\varphi_{V}^{tar},\varphi_{H}^{tar}\right) \end{array}\right.$$

where *f* is the process of calculating the sub-pixel coordinates corresponding to the target phase $\varphi_{V}^{tar}$ and $\varphi_{H}^{tar}$, using the coefficient matrices P and Q obtained in Equation (9), $u_{c}^{sp}$ and $v_{c}^{sp}$ are the sub-pixel level coordinates. As shown in Figure 4e, the three images on the left are zoomed-in images of the sub-pixel level feature points highlighted in the red rectangle box on the right images. It is obvious that the precision of the feature detection is improved.

The projector pixel coordinates corresponding to the feature points are

(11) $$\left\{\begin{array}{c} u_{p}=\frac{\varphi_{V}^{f}\left(u_{c}^{sp},v_{c}^{sp}\right)}{2\pi }T_{V}\;\\ v_{p}=\frac{\varphi_{H}^{f}\left(u_{c}^{sp},v_{c}^{sp}\right)}{2\pi }T_{H} \end{array}\right.$$

where $T_{V}$ and $T_{H}$ are the periods of fringe patterns along the vertical and horizontal directions, respectively, and $\varphi_{V}^{f}$ and $\varphi_{H}^{f}$ are the corresponding phases after being fitted with another polynomial in the same way as above.

### 3.3. World Coordinates Calculation

**Figure 5:**
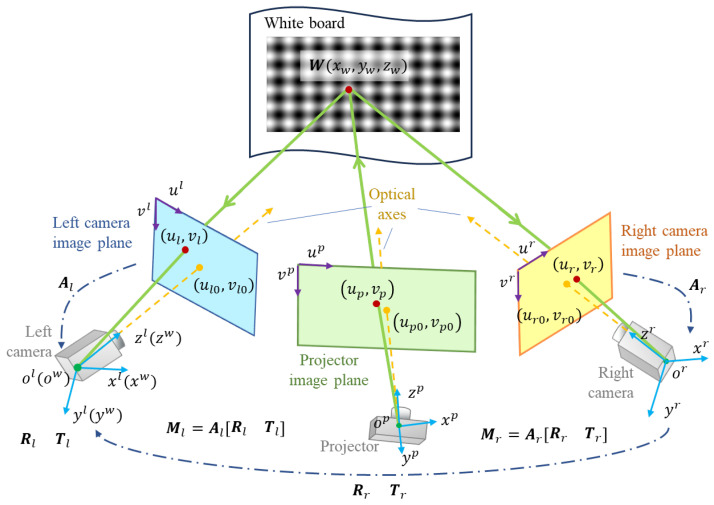
The relationship between projector and binocular camera.

When the left and right camera pixel coordinates $\left(u_{l},u_{r}\right)$ and $\left(v_{l},v_{r}\right)$ corresponding to the feature points are obtained, the world coordinates of the feature points can be calculated. As shown in Figure 5, let the projection matrix of the left camera be $M_{l}$, and the projection matrix of the right camera be $M_{r}$. After camera calibration, $M_{l}$ and $M_{r}$ are simplified to

(12) $$\left\{\begin{array}{c} M_{l}=\left[\begin{array}{cccc} 2760.8263 & 0 & 787.3906 & 0\\ 0 & 2761.8504 & 549.9437 & 0\\ 0 & 0 & 1 & 0 \end{array}\right]\;\\ M_{r}=\left[\begin{array}{cccc} 1956.2650 & -37.1332 & 2107.9197 & -864851.6178\\ -286.4883 & 2764.1705 & 527.0503 & 54260.4679\\ -0.5222 & -0.0080 & 0.8528 & 100.2849 \end{array}\right] \end{array}\right.$$

According to the projector model in Section 2.1, we have

(13) $$s_{l}\left[\begin{array}{c} u_{l}\\ v_{l}\\ 1 \end{array}\right]=A_{l}\left[R_{l}\;\;T_{l}\right]\left[\begin{array}{c} x^{w}\\ y^{w}\\ z^{w}\\ 1 \end{array}\right]=M_{l}\left[\begin{array}{c} x^{w}\\ y^{w}\\ z^{w}\\ 1 \end{array}\right]=\left[\begin{array}{cccc} m_{11}^{l} & m_{12}^{l} & m_{13}^{l} & m_{14}^{l}\\ m_{21}^{l} & m_{22}^{l} & m_{23}^{l} & m_{24}^{l}\\ m_{31}^{l} & m_{32}^{l} & m_{33}^{l} & m_{34}^{l} \end{array}\right]\left[\begin{array}{c} x^{w}\\ y^{w}\\ z^{w}\\ 1 \end{array}\right]$$

(14) $$s_{r}\left[\begin{array}{c} u_{r}\\ v_{r}\\ 1 \end{array}\right]=A_{r}\left[R_{r}\;\;T_{r}\right]\left[\begin{array}{c} x^{w}\\ y^{w}\\ z^{w}\\ 1 \end{array}\right]=M_{r}\left[\begin{array}{c} x^{w}\\ y^{w}\\ z^{w}\\ 1 \end{array}\right]=\left[\begin{array}{cccc} m_{11}^{r} & m_{12}^{r} & m_{13}^{r} & m_{14}^{r}\\ m_{21}^{r} & m_{22}^{r} & m_{23}^{r} & m_{24}^{r}\\ m_{31}^{r} & m_{32}^{r} & m_{33}^{r} & m_{34}^{r} \end{array}\right]\left[\begin{array}{c} x^{w}\\ y^{w}\\ z^{w}\\ 1 \end{array}\right]$$

Eliminate $s_{l}$ and $s_{r}$ in Equations (13) and (14), and then we have

(15) $$\left[\begin{array}{ccc} u_{l}m_{31}^{l}-m_{11}^{l} & u_{l}m_{32}^{l}-m_{12}^{l} & u_{l}m_{33}^{l}-m_{13}^{l}\\ v_{l}m_{31}^{l}-m_{21}^{l} & v_{l}m_{32}^{l}-m_{22}^{l} & v_{l}m_{31}^{l}-m_{23}^{l}\\ u_{r}m_{31}^{r}-m_{11}^{l} & u_{r}m_{32}^{r}-m_{12}^{r} & u_{r}m_{33}^{r}-m_{13}^{r}\\ v_{r}m_{31}^{r}-m_{21}^{l} & v_{r}m_{32}^{r}-m_{22}^{r} & v_{r}m_{33}^{r}-m_{23}^{r} \end{array}\right]\left[\begin{array}{c} x^{w}\\ y^{w}\\ z^{w} \end{array}\right]=\left[\begin{array}{c} m_{14}^{l}-u_{l}m_{34}^{l}\\ m_{24}^{l}-v_{l}m_{34}^{l}\\ m_{14}^{r}-u_{r}m_{34}^{r}\\ m_{24}^{r}-v_{r}m_{34}^{r} \end{array}\right]$$

The feature points’ coordinates of the three-dimensional world can be obtained by solving Equation (15). In this research, we assume that the origin of the left camera coordinate system $o^{l}-x^{l}y^{l}z^{l}$ coincides with the origin of the world coordinate system $o^{w}-x^{w}y^{w}z^{w}$ (i.e.,$\;x^{l}=x^{w}$,$\;y^{l}=y^{w}$,$z^{l}=z^{w}$). Therefore, we can obtain the coordinates of the feature points in the left camera coordinate system.

At this point, we have obtained the 3D coordinates of the feature point under the left camera and the sub-pixel level coordinates on the projector image plane. Next, the projector parameters can be optimized as shown in Section 3.4.

### 3.4. Projector Parameters Estimation

After determining the sub-pixel coordinates of the feature points on the projector image plane corresponding to the phase target as described in Section 2.2, all these point pairs are used to estimate the parameters of the projector. First, calculate the parameters without lens distortion through Zhang’s method. Then, nonlinear optimization is used to further solve the distortion parameters of the projector. All of the final parameters are optimized with the Levenberg–Marquardt optimization method by minimizing the reprojection error. The optimization objective function is given as follows

(16) $$F=\min\left(\sum_{i=1}^{n}\sum_{j}^{m}∥{w_{p}^{ij}-{\hat{w}}_{p}\left(A_{p},R_{p},T_{p},K_{p},W^{i}\right)∥}^{2}\right)$$

where *n* is the number of feature points on each phase target, *m* is the number of poses of the target, $w_{p}^{ij}$ is the pixel coordinate of the *j*-th point of the *i*-th pose on the image plane of the projector and ${\hat{w}}_{p}$ is the function representing the projection process of the projector. $A_{p}$, $R_{p}$, $T_{p}$, $K_{p}$ are the intrinsic matrix, rotation matrix, translation vector and the distortion coefficients of the projector, respectively. $W^{i}$ is the 3D coordinate of the feature points corresponding to $w_{p}^{ij}$, calculated by Equation (15).

Due to the assumption of Equation (12), the extrinsic parameters between the two cameras and the projector can be easily calculated. From this, the extrinsic parameters between each SCP can also be calculated.

## 4. Experiments and Results

**Figure 6:**
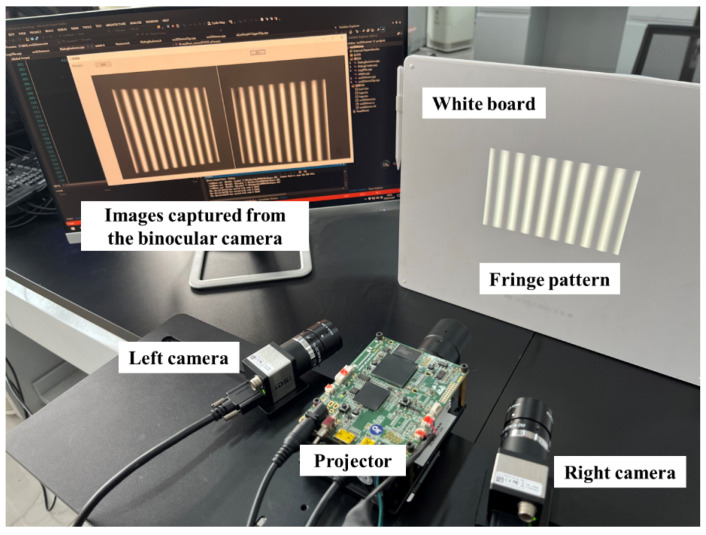
Experimental setup.

We set up a DCP system as shown in Figure 6 to test our algorithm. The system consists of two CMOS cameras with a resolution of 1600 × 1200 (model UI-3250CP-M-GL, produced by IDS Imaging Development Systems GmbH, Obersulm, Germany). The DLP projector with a projection speed of 30 fps and a resolution of 1280 × 800 (model PDC03-A, produced by Giant Vinda, Fuzhou, China). The focal length of the lens of both cameras is 12 mm (Japan Ricoh Corporation, FL-CC1214-2M, Tokyo, Japan). For algorithm validation, we calibrated the system using both the classical separate calibration method and our coupled calibration method. For both methods, we used $T_{V}=80$ pixels and $T_{H}=50$ pixels per period of fringe patterns and N=8 steps phase shifting for calibration.

After the fringe images are acquired by the experimental setup shown in Figure 6, and processed by Figure 4a,b, the feature point can be extracted. We conduct a comparative experiment between the pixel level feature point extraction method and our sub-pixel level extraction method with polynomial fitting. The MSE (Mean Squared Error) between pixel level points and sub-pixel level points is 0.2034 pixels.

**Figure 7:**
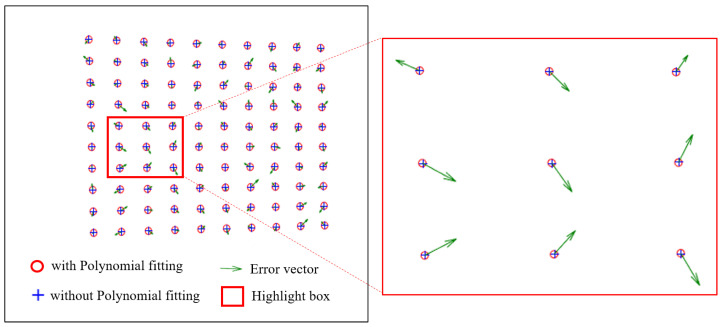
The comparison results of polynomial fitting and general feature point extraction methods.

Figure 7 shows the comparison results of the polynomial fitting method and the general method to extract feature points. The red circle indicates the sub-pixel level coordinates refined by the polynomial fitting method, corresponding to Figure 4d,e. The blue cross indicates the original pixel level coordinates calculated based on phase value only, without being optimized by the polynomial fitting method, and corresponding to Figure 4c. The green arrow indicates the error vector between the sub-pixel accuracy value and the pixel accuracy value. The highlighted box shows the zoomed-in comparison. It can be clearly seen that the polynomial fitting method can avoid the nonlinear errors caused by the overall tilt and subtle deformation of the white board.

The intrinsic parameters and distortion coefficients of the projector are calibrated with our proposed method and classical method, respectively. It is worth mentioning that both Left-SCP and Right-SCP can be used for projector calibration when the classical method is used. The calibration results are listed in Table 1 and Table 2. It is obvious that the standard errors of the calibration results with our method are much lower than those obtained with the classical method.

**Figure 8:**
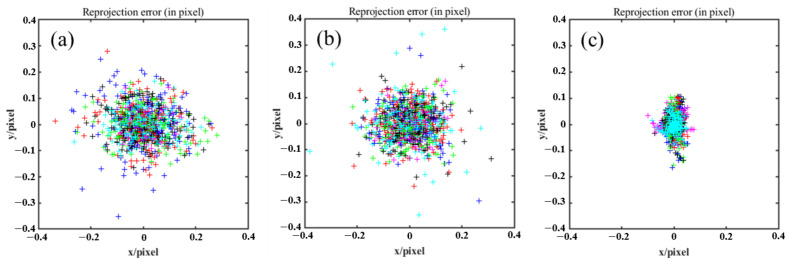
Reprojection error distributions. (**a**) Separate calibration method (Left-SCP). (**b**) Separate calibration method (Right-SCP). (**c**) Coupled calibration method (ours).

As shown in Figure 8, to evaluate the calibrated intrinsic parameters, the reprojection errors are calculated for every plane orientation, while every color expresses one of the planes’ orientations. The reprojection error distributions of the separate calibration methods are shown in Figure 8a and Figure 8b, respectively, while Figure 8c shows our method. The reprojection errors of the Left-SCP and the Right-SCP calibrated by classical method are (0.0836, 0.0675) and (0.0853, 0.0718), respectively. Our proposed method reduces these figures to (0.0186, 0.0322). Such a significant improvement is not only mainly caused by the accumulation of the calibration error of the camera, but also the extraction error and phase error.

**Figure 9:**
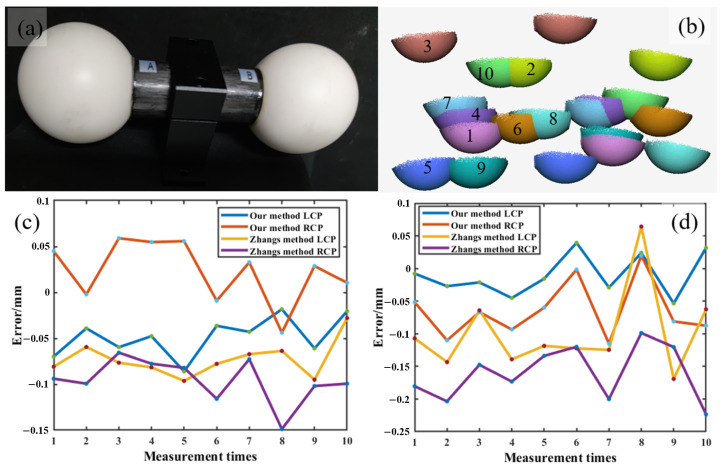
Comparison of ceramic sphere measurements. (**a**) Measured ceramic spheres. (**b**) 3D point cloud at different locations. (**c**) Measurement results of sphere A. (**d**) Measurement results of sphere B.

We measured two ceramic spheres to test the accuracy of our algorithm. To show that our calibration method indeed reconstructs the absolute 3D geometry, we measured the sphere using both our coupled calibration method and the classical separate calibration method. In this experiment, as shown in Figure 9a, two ceramic spheres with diameters of 50.7991 mm and 50.7970 mm were measured ten times from different views. Figure 9b shows the 3D point clouds, with ten measurements, and the different numbers represent the measurement results at each position. By fitting two spheres using the 3D point cloud, the diameters of the two spheres can be obtained. The measurement results of sphere A and sphere B are shown in Figure 9c and Figure 9d, respectively. The Mean Absolute Error (MAE) of the results, measured ten times, using three methods are calculated and shown in Table 3. In Table 3, the measurement accuracy of the proposed method can achieve a spatial resolution of 0.07 mm, which is more accurate than the classical method.

## 5. Discussion

In this study, we proposed a new method for calibrating projector parameters in a DCP system with high accuracy. In some difficult measurement environments, the projector in the DCP system is seen as an inverse camera, thus playing the role of providing both texture and 3D information. In many previous methods [15,16,17,18], the projector parameters are calibrated in the Left-SCP and Right-SCP, respectively; thus the projector parameters were inconsistent in the two systems due to influencing factors such as camera calibration error transmission and feature point extraction error that may occur in the calibration process, which led to measurement errors. Even though some learning-based calibration methods have emerged [22,23], methods for calibrating the entire DCP system simultaneously are still lacking. Differently from other methods, in order to unify the projector parameters in the whole DCP system, we innovatively proposed a coupled calibration method, which uses the binocular camera to uniquely determine the coordinates of 3D feature points. At the same time, we also used a combination of phase target and polynomial fitting to obtain the coordinates of the feature points at a sub-pixel level, thus simplifying the procedure.

## 6. Conclusions

We develop a novel projector calibration framework based on binocular structured light systems. Through the binocular structured light system and the phase target, the inconsistency of the calibration results between the Left-SCP and the Right-SCP in the traditional structured light system is effectively eliminated. The experimental results show that the average reprojection error of the proposed method can reach (0.0186, 0.0322) pixels. Specifically, we achieved an average accuracy of 0.07 mm by repeatedly measuring two standard spherical objects. The experimental results are significantly better than the traditional methods.

## Figures and Tables

**Table 1:** Calibrated intrinsic parameters with standard error (unit: pixel).

Method	Device	$f_{\mathrm{up}}$	$f_{\mathrm{vp}}$	$u_{p0}$	$v_{p0}$
Separate calibration	Left-SCP	1744.3991 ± 16.9957	1745.2484 ± 16.9660	588.1513 ± 3.3219	375.4900 ± 3.9784
(Classical)	Right-SCP	1755.7047 ± 18.4074	1755.2361 ± 18.3390	597.7887 ± 3.3325	366.9479 ± 4.3530
Coupled calibration (Ours)	DCP	1756.5209 ± 0.7293	1756.2796 ± 0.7163	597.7667 ± 0.3391	382.3472 ± 0.3184

**Table 2:** Calibrated distortion coefficients with standard error.

Method	Device	$k_{p1}$	$k_{p2}$	$p_{p1}$	$p_{p2}$
Separate calibration	Left-SCP	−0.0718 ± 0.0300	0.3390 ± 1.2365	0.0002 ± 0.0004	−0.0042 ± 0.0006
(Classical)	Right-SCP	−0.0762 ± 0.0287	−0.2317 ± 1.1333	−0.0018 ± 0.0004	−0.0014 ± 0.0006
Coupled calibration (Ours)	DCP	−0.0610 ± 0.0024	0.0223 ± 0.0461	−0.0001 ± 0.00004	−0.0014 ± 0.00005

**Table 3:** Comparison of the MAE of the proposed method and the conventional methods (unit: mm).

Method	Device	MAE of the Diameter of Sphere A	MAE of the Diameter of Sphere B
Separate calibration	Left-SCP	0.0726	0.1115
(Classical)	Right-SCP	0.0957	0.1601
Coupled calibration	Left-SCP	0.0481	0.0294
(Ours)	Right-SCP	0.0341	0.0687

## Data Availability

Data are contained within the article.

## Abbreviations

The following abbreviations are used in this manuscript:

SCP	Single Camera-Projector
DCP	Dual Cameras-Projector
